# The association between local brain structure and disgust propensity

**DOI:** 10.1038/s41598-022-05407-4

**Published:** 2022-01-25

**Authors:** Albert Wabnegger, Carina Schlintl, Anne Schienle

**Affiliations:** 1 Department of Clinical Psychology, University of Graz, Universitätsplatz 2/DG, 8010 Graz, Austria; 2 BioTechMed, Graz, Austria

**Keywords:** Neuroscience, Emotion, Insula

## Abstract

Research has discovered structural differences in the brains of people with different personality types. In the present voxel-based morphometry study we focused on the association between disgust propensity (DP: the temporally stable tendency to experience disgust across different situations) and grey matter volume (GMV) in regions of interest [insula, orbitofrontal cortex (OFC), basal ganglia]. We collected structural brain scans from 498 healthy individuals (352 females, 146 males; mean age = 27 years). Regression analyses were performed to test the association between three domains of DP (core, animal-reminder, contamination) and GMV. We observed negative correlations between animal-reminder DP and the volume of the insula, and contamination DP and OFC volume. Animal-reminder DP correlated positively with GMV in the basal ganglia (putamen). This study identified weak correlations between local brain volume and disgust propensity. The association between DP and insula volume concerned the posterior insula and was in the opposite of the expected direction. The findings of this study are inconsistent with the concept of the anterior insula as a region that specifically mediates DP.

## Introduction

Disgust propensity (DP) is a personality trait that describes the general tendency of a person to respond with the emotion of disgust to different stimuli and situations^1–4^. Disgust elicitors include food with bad taste (e.g., bitter/spoiled/poisonous substances), poor hygiene, certain animals (e.g., cockroaches), stimuli that signal disease (e.g., skin abnormalities), and social/moral transgressions (e.g., cheating, incest). Some researchers have claimed that disgust evolved from a ‘bitter taste rejection mechanism’ that functions to protect us from the oral incorporation of contaminants^3^. During biological and cultural evolution, the eliciting categories increased to comprise not only health-threatening stimuli but also those that endanger social and moral systems.

The most widely used measure of DP is the Disgust Scale^5^ and its adaptations in various languages. Studies on the factor structure of DP have repeatedly identified three dimensions across different countries (e.g., Australia, Brazil, Germany, Italy, Japan, Sweden, and United States) in large representative samples^6,7^. The three DP categories are labeled Core Disgust (e.g., drinking spoiled milk), Animal-Reminder Disgust (e.g., walking through a graveyard), and Contamination Disgust (e.g., touching the toilet seat in a public restroom). These reliable and valid DP dimensions reflect disgust triggered by the threat of disease through mostly oral contact with pathogens (core), reminders of one’s mortality (animal-reminder), and non-oral contact with infectious agents (contamination). Females generally score higher on the three disgust dimensions than males^6,7^.

A multitude of brain imaging investigations has focused on the neural correlates of disgust. The findings have been combined in meta-analyses on the functional neuroanatomy of basic emotions. Some of these analyses were based on a ‘locationist approach’ and provided evidence for distinct neural correlates of disgust^8–10^. Feeling disgusted was specifically correlated with increased activity in the insula, the basal ganglia, and frontal brain regions, such as the dorsolateral prefrontal cortex (DLPFC) and orbitofrontal cortex (OFC). Other authors^11^ have followed a ‘constructionist approach’, which assumes that emotions are built from more basic neural processes. In their meta-analysis, Lindquist et al.^11^ also identified the insula and prefrontal regions as central for emotion processing; however, across all basic emotion categories. According to this view, the insula is involved in interoception and the awareness of affective feelings. Frontal brain areas use stored representations of prior experiences to make meaning of core affective inputs (mental representation of bodily sensations that can be experienced as feelings of (dis)pleasure).

To the best of our knowledge, there is only one study on the structural neuroanatomy of DP with a relatively small sample size (n = 99 females)^12^. In this study, food-related DP (disgust-related oral rejection) was positively correlated with grey matter volume (GMV) in the anterior insula, while DP concerning contact with decaying and dead organisms correlated negatively with GMV in the dorsomedial/ lateral prefrontal cortex.

The goal of the present voxel-based morphometry (VBM) study was to identify neural correlates of three reliable dimensions of DP (core/animal-reminder/contamination) within a large sample of healthy males and females (n = 498). We used a region-of-interest approach, where we correlated GMV in specific brain areas (insula, putamen, OFC, DLPFC) with scores obtained on the Questionnaire for the Assessment of Disgust Propensity^13^. Based on the mentioned previous findings^8–10,12^, we expected positive associations between DP and GMV in the insula and putamen, as well as negative associations between DP and GMV in the OFC/DLPFC.

## Results

### Self-reports

Descriptive statistics for the Questionnaire for the Assessment of Disgust Propensity (QADP; scores for the subscales/total score) and sex-group comparisons are depicted in Table 1. Females scored higher on the QADP than males (all p < 0.001). Correlations between the three QADP subscales were as follows: animal-reminder/core: r = 0.62; core/contamination: r = 0.78, contamination/animal-reminder: r = 0.58 (all p < 0.001). Age was negatively associated with animal-reminder DP (r: − 0.24) and contamination DP (r: − 0.14; both p < 0.001).

**Table 1:** Questionnaire for the assessment of disgust propensity (QADP): descriptive statistics for disgust domains and comparisons of males and females.

Domains	M (SD) [95% CI] all (n = 498)	Range	M (SD) [95% CI] female (n = 352)	M (SD) [95%CI] males (n = 146)	T_224–300_ (p)
Animal-Reminder	1.43 (0.91) [1.35–1.52]	0.00–3.78	1.62 (0.89) [1.53–1.71]	0.98 (0.80) [0.86–1.11]	7.50 (< 0.001)
Contamination	1.84 (0.71) [1.78–1.91]	0.15–3.69	1.99 (0.64) [1.93–2.05]	1.48 (0.73) [1.36–1.61]	7.73 (< 0.001)
Core	2.64 (0.70) [2.57–2.70]	0.60–4.00	2.80 (0.60) [2.74–2.86]	2.23 (0.76) [2.10–2.36]	8.96 (< 0.001)
Total QADP score	2.07 (0.67) [2.01–2.13]	0.35–3.74	2.24 (0.59) [2.18–2.30]	1.66 (0.68) [1.55–1.77]	9.57 (< 0.001)

Confidence intervals are bias-corrected and accelerated (1000 samples).

### Voxel-based morphometry

#### Region-of-interest analyses

**Figure 1:**
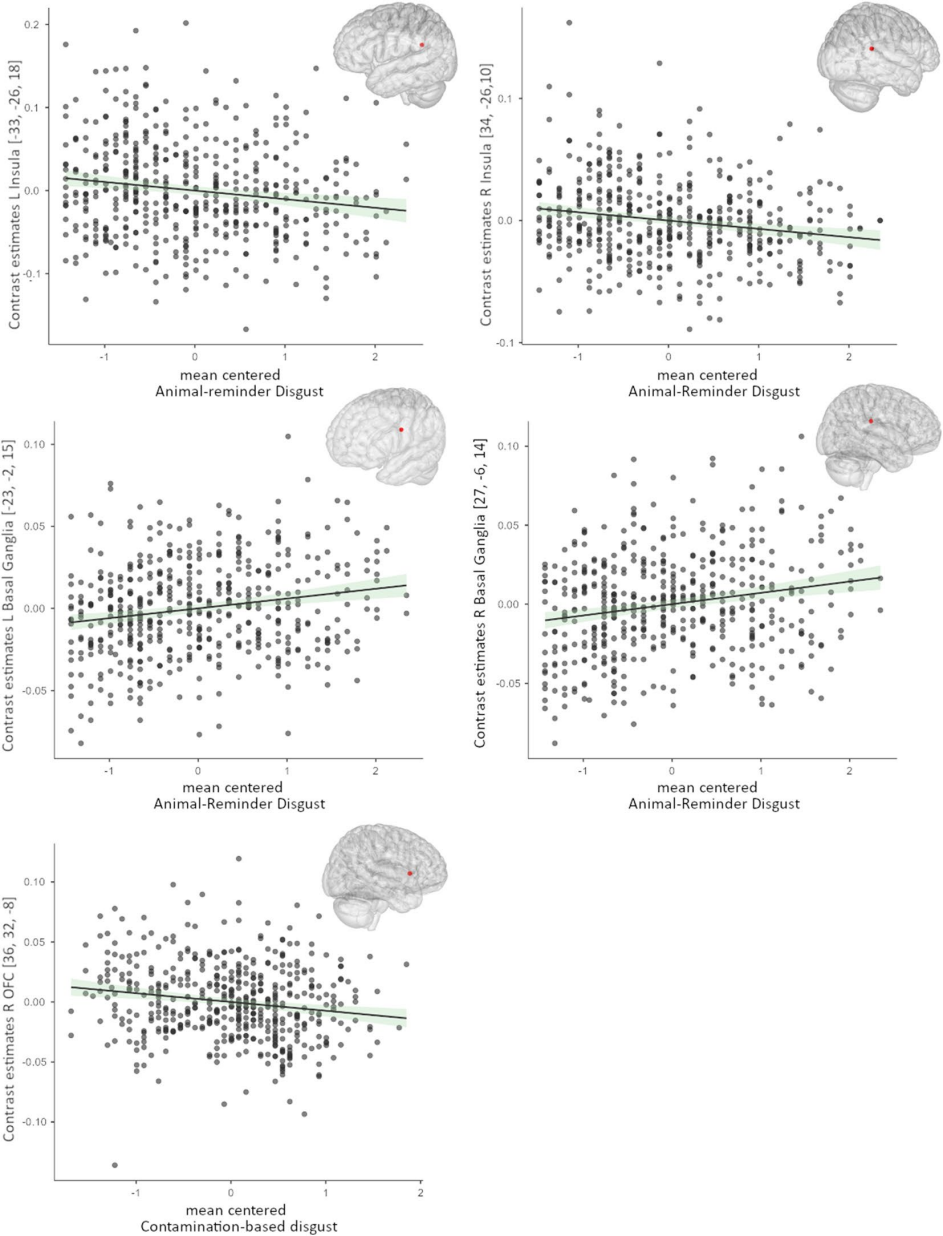
Correlations (standard error) between disgust propensity (animal-reminder, contamination) and volume in the left/right insula, left/right basal ganglia, and right orbitofrontal cortex.

Animal-reminder DP was negatively correlated with GMV in the bilateral posterior insula and positively with GMV in the bilateral basal ganglia (peak: putamen; Table 2). Contamination DP was negatively associated with GMV in the right orbitofrontal cortex (Fig. 1). The total QAPD score was not significantly correlated with GMV in the selected ROIs. (For all non-significant correlations between DP and GMV see Supplementary Table 1).

**Table 2:** Correlations between grey matter volume and disgust propensity.

Domain	ROI	H	X	Y	Z	T	P (FWE)	Cohen’s d
Animal− Reminder+	Basal ganglia	L	− 23	− 2	15	3.86	0.011	0.15
Animal− Reminder+	Basal ganglia	R	27	− 6	14	4.16	0.003	0.25
Animal− Reminder−	Insula	R	34	− 26	10	3.82	0.008	0.30
Animal− Reminder−	Insula	L	− 33	− 26	18	3.28	0.046	0.30
Contamination−	OFC	R	36	32	− 8	3.34	0.037	0.26
**Contamination−**	**Fusiform gyrus**	**L**	− **34**	− **74**	− **12**	**4.84**	**0.014**	**0.43**

+/− positive/negative correlations corrected for family-wise error (FWE), *H* hemisphere, MNI coordinates (x, y, z), *OFC* orbitofrontal cortex, bold whole-brain results.

#### Whole-brain analysis

Contamination DP correlated negatively with GMV in the left fusiform gyrus (MNI x, y, z: − 34, − 74, − 12, t-value: 4.84, p(FWE) = 0.014, Cohen’s d: 0.43).

Based on a reviewer’s suggestion, we examined possible interaction effects between sex and DP on GMV (whole-brain analysis). Female participants showed a positive association between contamination DP and GMV in the left cerebellum (crus 2; MNI x, y, z: − 38, − 14, 9, t-value: 4.76, p(FWE) = 0.02; Cohen’s d: 0.15), while males showed an inverse association. The other QADP subscales and the total score showed no sex-specific associations with GMV.

### Surface-based morphometry

Based on a reviewer’s suggestion, we investigated the association between surface-based morphometry and DP. The conducted whole-brain analysis revealed no significant (all ps ≥ 0.08) association between any of the four DP scales (animal reminder, contamination, core disgust, overall disgust), cortical thickness, gyrification, and sulcal depth (see Supplementary Table 2).

## Discussion

This VBM study investigated correlations between three reliable domains of disgust propensity (core, animal-reminder, contamination) and GMV in specific regions of the brain (insula, pallidum, OFC, DLPFC). The results are based on a large data set consisting of structural brain scans from 498 healthy individuals of both sexes.

We predicted a positive association between GMV in the anterior insula and DP because according to a locationist account, this region is the neural substrate of disgust^14^. However, in the present study, none of the DP domains was related to GMV in the anterior insula. Instead, animal-reminder DP correlated negatively with GMV in the posterior insula (PI).

Based on the results of their meta-analysis, Kurth et al.^27^, identified four functionally distinct regions in the human insula: two anterior regions (with cognitive/affective functions), a central olfactory-gustatory region, and a posterior sensorimotor region. The PI, which is connected to somatosensory and motor cortices, is responsive to painful, visceral, and somatosensory stimulation. Direct electrical stimulation of this region in humans has provoked interoceptive and somatic sensations (e.g., changes in gastric motility; for a review see^27^). Underlining the motor functions of the PI, Gerlach et al.^28^ proposed that the PI can shift behavioral strategies upon the detection of aversive internal states. In this sense, the neural correlate of DP involved regions with functions such as homeostasis/allostasis and the selection of adaptive response strategies^29^.

We observed a positive correlation between animal-reminder DP and bilateral basal ganglia volume. The peak was located in the putamen. A highly-cited case study by Calder et al.^15^ identified impaired recognition and experience of disgust in a patient with damage of the putamen and the insula. Deficits in disgust processing have also been observed In patients with Huntington’s disease, which is associated with striatal neurodegeneration^30^. Imaging studies with healthy individuals showed that disgust elicitation (internally via imagery and externally via aversive smells) was accompanied by putamen activation^16,17^. Moreover, two studies^18,19^ found that disgust propensity predicted pallidal (and insular) responses to disgusting pictures (e.g., disgusting food).

Finally, we looked at correlations between DLPFC/OFC volume and DP. We replicated a previous finding of a negative association between OFC volume and DP^12^. OFC activity has been repeatedly observed during disgust processing^20,21^, and disgust-related OFC activity has been correlated with DP^22^. Nevertheless, the emotion specificity of these findings can be questioned since recruitment of the OFC (and putamen) also occurs during the experience of other basic emotions^11,20^.

The whole-brain approach revealed an unexpected finding: DP was associated with GMV in the fusiform gyrus. This region entails higher processing of visual information, such as identification and differentiation of objects (e.g., face/body recognition, identification of features within a category). In addition, the fusiform gyrus is involved in multisensory integration and memory^31^. Although it seems understandable that the mentioned functions could be relevant for disgust processing (e.g., recognition/ differentiation of contaminants), this aspect requires further investigation.

Replicating previous findings, we observed sex differences regarding self-reports for DP^7,13^. Females scored higher on all three subscales of the QADP (core, animal-reminder, contamination) than males. Based on an evolutionary approach, it has been argued that the disease-avoidance emotion of disgust is more pronounced in females because they play a double role in protecting both self and offspring from infectious disease^32^. Curtis et al.^32^ conducted a large web-based survey with over 40,000 individuals. The participants viewed images with high versus low disease relevance and evaluated the intensity of elicited disgust (1–5; 5 = very high). Disease-salient images were rated as more disgusting by females (M = 3.5) than males (M = 3.2). This difference was statistically significant (p < 0.001); however, the practical significance of this small difference for disgust-motivated behavior in everyday life seems questionable. In an fMRI experiment on the processing of disgusting images^33^, sex differences in reported disgust were also identified but the brain activation did not differ between males and females. These findings indicate that sex differences in disgust processing are smaller than previously assumed.

The analysis of the brain-structural data focusing on sex differences revealed a positive association between contamination DP and GMV in the left cerebellum in female participants and a negative association in males. Previous research has shown that brain structure-personality relationships can differ between the sexes^34,35^. Moreover, disgust-related functions have been repeatedly identified for the cerebellum^36^, which forms a subcortical integration hub for motor/affective processes.

In conclusion, we observed modest correlations between disgust propensity and local brain volume. The correlations were partly not in the expected direction (e.g., negative correlation between insula GMV and DP) and not present for the expected domains (no correlation between core DP and insula GMV).

Recent studies on the structural neuroanatomy of personality have revealed similar findings. For example, a large study with more than 1000 participants identified no correlation between the Big-Five dimensions of personality and brain volume, except for neuroticism^23^. Very similar, Avinum et al.^24^ found little evidence for associations between the Big-Five traits and variability in brain grey or white matter (n = 1107 university students). The authors argued that small sample sizes and the heterogeneous methodology of previous studies were the reasons for the discrepant findings. Within this context, Kharabian Masouleh et al.^25^ advised investigating samples of more than 300 participants to be able to reliably detect associations between psychological phenotypes and brain morphometry.

We have to mention the following limitations of the present study. This study focused on DP but did not consider or control for non-target personality traits. For example, a person high in DP might score high (or low) on other traits. Thus, patterns of personality traits might be a more adequate construct to study instead of single traits. Following this idea, neural correlates of personality may be more readily identified in functional network measures, such as functional connectivity. Moreover, multivariate statistical methods that jointly model several brain regions and DP domains could help to decrease the number of computed analyses. We studied a self-selected sample of predominantly university students. The present findings may therefore not be representative of the general population. Finally, the sample was unbalanced for gender.

## Method

### Participants

Structural brain data from 498 individuals (352 females, 146 males; all Caucasian) with a mean age of 27.37 years (SD = 9.34) were analyzed. The majority of the participants had obtained at least a high-school diploma (72%) and were university students. Exclusion criteria for participation were reported pregnancy, metal or electronic implants (e.g., surgical devices, metallic tattoos on the head), a history of trauma/fainting, reported major medical, neurological, and mental disorders (e.g., diagnoses of tumors, heart conditions, affective disorders, claustrophobia), as well as medication (agents known to affect brain function, such as psychotropic medication).

The data had been collected across three scanner sites (see Supplementary Table 3). All participants provided written informed consent. The data acquisition was conducted following the Declaration of Helsinki and had been approved by the ethics committees that were responsible at the scanner sites (ethics committee of the German Society for Psychology; ethics committee of the University of Graz; ethics committee of the Medical University of Graz).

### Disgust propensity

Disgust propensity (DP) was assessed with the Questionnaire for the Assessment of Disgust Propensity (QADP^13^). This questionnaire has 37 items, which are answered on 5-point scales (0 = not disgusting; 4 = very disgusting). The QADP was evaluated in a representative sample of n = 2473 healthy individuals^7^. The conducted exploratory/confirmatory factor analyses identified three interrelated dimensions: Animal-Reminder Disgust (9 items; e.g., ‘you touch a skull’), Core Disgust (15 items; e.g., ‘you drink spoiled milk’), and Contamination Disgust (13 items; e.g., ‘you touch the toilet seat in a public restroom’). In the present investigation (n = 498), all QADS scales had good reliabilities (McDonald’s omega (ω); Core: 0.88; Animal-Reminder: 0.87; Contamination: 0.83). Additionally, we calculated a composite score of all three subscales to receive a measurement of overall disgust propensity (ω = 0.93).

### MRI recording

Structural T1-weighted images were assessed with three different MRI scanners (1.5 T Siemens Symphony, 3 T Siemens Skyra, 3 T Siemens Tim Trio). Detailed information about scanner settings can be found in the supplementary material (Supplementary Table 3). The structural scans were analyzed with Matlab R2019b and the Computational Anatomy Toolbox (CAT12; v1742; http://www.neuro.uni-jena.de/cat/) implemented in SPM12 (v7771; Wellcome Trust Centre for Neuroimaging; http://www.fil.ion.ucl.ac.uk/spm/software/spm12/) to gain voxel-wise comparisons of grey matter volume (GMV). Structural data were segmented into grey matter, white matter, and cerebrospinal fluid. Spatial registration of grey matter images was carried out by using the optimized shooting approach^26^. To preserve the total amount of grey matter, signal images were modulated. The final resulting voxel size was 1.5 × 1.5 × 1.5 mm. Segmented grey matter images were smoothed with a Gaussian kernel with a full width at half maximum (FWHM) of 8 mm. Finally, only voxels with a grey matter volume of at least 0.1 were analyzed (absolute threshold).

### Morphological analysis

#### Voxel-based morphometry (VBM)

We conducted whole-brain analyses as well as region of interest (ROI) analyses. Based on previous research on disgust propensity^12^ and neurofunctional correlates of disgust^8–10^, we selected the following ROIs: insula, basal ganglia (caudate, pallidum, putamen), orbitofrontal cortex (OFC), and dorsolateral prefrontal cortex (DLPFC). Masks were derived from the Harvard–Oxford atlas.

#### Surface-based morphometry (SBM)

Based on a reviewer’s suggestion, we calculated SBM analyses (gyrification index, thickness, and sulcal depth) by using the default settings of the CAT12-toolbox. Thickness data were smoothed with a value of 15 mm, and folding data (gyrification/depth) were smoothed with a value of 20 mm as recommended by the cat12-manual.

### Statistical analyses

QADP scores were compared between males and females via t-tests (Levene). For the GMV data, multiple regression analyses were computed. We investigated associations between the mean-centered QADP scales (core, animal-reminder, contamination, total score) and GMV in the defined ROIs (insula, basal ganglia, OFC, DLPFC) in four separate general linear models due to multicollinearity of the subscales. Further, we set up full-factorial models to investigate the interaction between sex and disgust propensity (QADP scales) on GMV (based on the suggestions of a reviewer). The following covariates were used in the analyses: total intracranial volume, image quality index provided by the CAT toolbox, scanner, sex, and age. Results from the whole-brain analysis and ROI results (with small volume correction) were considered significant if the peak-level statistic was below p < 0.05, corrected for family-wise error (FWE).

## Supplementary Information

Supplementary Table 1.

Supplementary Table 2.

Supplementary Table 3.

## Data Availability

The dataset generated during and/or analyzed during the current study is available from the corresponding author on reasonable request.
