# Supplementary material for: The association between local brain structure and disgust propensity
Source: Sci Rep. 2022 Jan 25;12:1327. doi: 10.1038/s41598-022-05407-4 (PMC8789785; doi:10.1038/s41598-022-05407-4)
Supplement: Supplementary file 1 — Supplementary Table 1. [file 41598_2022_5407_MOESM1_ESM.docx]

**Supplementary Table 1: Non-significant (p >.05) correlations between grey matter volume in regions of interest and disgust propensity**

| **Domain** | **ROI** | **H** | **X** | **Y** | **Z** | **T** | **p_FWE-corr** | **Cohen´s d** |
| --- | --- | --- | --- | --- | --- | --- | --- | --- |
|  |  |  |  |  |  |  |  |  |
| Animal-Reminder+ | DLPFC | L | -40 | 34 | 39 | 2.48 | .663 | .22 |
| Animal-Reminder+ | DLPFC | R | 28 | -2 | 56 | 3.00 | .284 | .26 |
| Animal-Reminder+ | Insula | L | -36 | -9 | -6 | 1.78 | .687 | .16 |
| Animal-Reminder+ | Insula | R | No suprathreshold clusters | | | | | |
| Animal-Reminder+ | OFC | L | -8 | 36 | -24 | 1.85 | .675 | .15 |
| Animal-Reminder+ | OFC | R | 15 | 33 | -26 | 2.36 | .328 | .21 |
| Animal-Reminder+ | Putamen | R | 27 | -6 | 14 | 4.16 | .186 | .25 |
| Animal-Reminder- | Basal ganglia | L | No suprathreshold clusters | | | | | |
| Animal-Reminder- | Basal ganglia | R | 32 | -20 | 9 | 1.92 | .737 | .22 |
| Animal-Reminder- | DLPFC | L | -36 | 10 | 39 | 2.49 | .657 | .22 |
| Animal-Reminder- | DLPFC | R | 18 | 22 | 51 | 2.51 | .649 | .22 |
| Animal-Reminder- | OFC | L | -42 | 32 | -6 | 1.93 | .631 | .17 |
| Animal-Reminder- | OFC | R | 28 | 27 | -26 | 1.90 | .577 | .15 |
|  |  |  |  |  |  |  |  |  |
| Contamination+ | Basal ganglia | L | No suprathreshold clusters | | | | | |
| Contamination+ | Basal ganglia | R | No suprathreshold clusters | | | | | |
| Contamination+ | DLPFC | L | -30 | 16 | 44 | 2.16 | .853 | .15 |
| Contamination+ | DLPFC | R | 38 | -4 | 46 | 2.19 | .850 | .16 |
| Contamination+ | Insula | L | No suprathreshold clusters | | | | | |
| Contamination+ | Insula | R | No suprathreshold clusters | | | | | |
| Contamination+ | OFC | L | No suprathreshold clusters | | | | | |
| Contamination+ | OFC | R | No suprathreshold clusters | | | | | |
|  |  |  |  |  |  |  |  |  |
| Contamination- | Basal ganglia | L | No suprathreshold clusters | | | | | |
| Contamination- | Basal ganglia | R | No suprathreshold clusters | | | | | |
| Contamination- | DLPFC | L | -30 | 2 | 54 | 2.29 | .784 | .20 |
| Contamination- | DLPFC | R | 27 | 32 | 40 | 2.05 | .905 | .18 |
| Contamination- | Insula | L | No suprathreshold clusters | | | | | |
| Contamination- | Insula | R | 46 | -2 | -3 | 2.87 | .130 | .25 |
| Contamination- | OFC | L | -22 | 18 | -24 | 2.77 | .181 | .25 |
|  |  |  |  |  |  |  |  |  |
| Core Disgust+ | Basal ganglia | L | -24 | -2 | 15 | 2.45 | .397 | .15 |
| Core Disgust+ | Basal ganglia | R | 24 | 2 | 12 | 1.98 | .701 | .18 |
| Core Disgust+ | DLPFC | L | -38 | 21 | 32 | 2.40 | .720 | .21 |
| Core Disgust+ | DLPFC | R | 38 | -4 | 46 | 2.18 | .852 | .16 |
| Core Disgust+ | Insula | L | -42 | 10 | -3 | 1.67 | .741 | .15 |
| Core Disgust+ | Insula | R | No suprathreshold clusters | | | | | |
| Core Disgust+ | OFC | L | No suprathreshold clusters | | | | | |
| Core Disgust+ | OFC | R | No suprathreshold clusters | | | | | |
| Core Disgust - | Basal ganglia | L | No suprathreshold clusters | | | | | |
| Core Disgust - | Basal ganglia | R | No suprathreshold clusters | | | | | |
| Core Disgust - | DLPFC | L | -4 | -3 | 70 | 2.34 | .755 | .22 |
| Core Disgust - | DLPFC | R | 28 | 30 | 39 | 1.69 | .978 | .15 |
| Core Disgust - | Insula | L | -33 | -18 | 21 | 2.37 | .356 | .19 |
| Core Disgust - | Insula | R | 46 | -3 | -3 | 2.74 | .172 | .23 |
| Core Disgust - | OFC | L | -22 | 20 | -26 | 2.09 | .544 | .19 |
| Core Disgust - | OFC | R | 24 | 21 | -18 | 2.60 | .216 | .23 |
|  |  |  |  |  |  |  |  |  |
| Total+ | Basal ganglia | L | -24 | -2 | 15 | 3.00 | .133 | .15 |
| Total+ | Basal ganglia | R | 27 | 0 | 15 | 2.55 | .337 | .15 |
| Total+ | DLPFC | L | -39 | 21 | 32 | 2.31 | .772 | .21 |
| Total+ | DLPFC | R | 30 | -2 | 54 | 2.58 | .592 | .15 |
| Total+ | Insula | L | No suprathreshold clusters | | | | | |
| Total+ | Insula | R | No suprathreshold clusters | | | | | |
| Total+ | OFC | L | No suprathreshold clusters | | | | | |
| Total+ | OFC | R | No suprathreshold clusters | | | | | |
| Total- | Basal ganglia | L | No suprathreshold clusters | | | | | |
| Total- | Basal ganglia | R | No suprathreshold clusters | | | | | |
| Total- | DLPFC | L | -38 | 9 | 40 | 2.00 | .916 | .18 |
| Total- | DLPFC | R | 18 | 21 | 52 | 2.14 | .870 | .19 |
| Total- | Insula | L | -33 | -26 | 18 | 2.41 | .331 | .22 |
| Total- | Insula | R | 46 | -3 | -4 | 3.09 | .075 | .26 |
| Total- | OFC | L | -22 | 20 | -26 | 2.05 | .563 | .19 |
| Total- | OFC | R | 36 | 32 | -6 | 2.84 | .131 | .24 |

Footnote: +/-: positive/ negative correlations; H = hemisphere; MNI coordinates (x,y,z); OFC: orbitofrontal cortex; DLPF: dorsolater prefrontal cortex
