# Supplementary material for: The association between local brain structure and disgust propensity
Source: Sci Rep. 2022 Jan 25;12:1327. doi: 10.1038/s41598-022-05407-4 (PMC8789785; doi:10.1038/s41598-022-05407-4)
Supplement: Supplementary file 2 — Supplementary Table 2. [file 41598_2022_5407_MOESM2_ESM.docx]

**Supplementary Table 2a: Non-significant (p >.05) whole-brain correlations between sulcal depth and domains of disgust propensity**

| **Domain** | **Region** | **H** | **X** | **Y** | **Z** | **T** | **p_FWE-corr** |
| --- | --- | --- | --- | --- | --- | --- | --- |
| Animal-Reminder+ | Precentral | R | 29 | -9 | 57 | 3.59 | .416 |
| Animal-Reminder- | Superior Frontal, orbital | L | -30 | 58 | -2 | 3.64 | .367 |
|  |  |  |  |  |  |  |  |
| Core-Disgust+ | Middle Frontal | R | 31 | 41 | 24 | 3.63 | .367 |
| Core-Disgust- | Middle Temporal | L | -63 | -29 | -15 | 3.54 | .471 |
|  |  |  |  |  |  |  |  |
| Contamination+ | Occipital inferior | L | -43 | -71 | -8 | 3.67 | .343 |
| Contamination- | Fusiform | L | -36 | -76 | -17 | 4.00 | .123 |
|  |  |  |  |  |  |  |  |
| Total+ | Middle Temporal | L | -42 | -58 | 13 | 3.35 | .672 |
| Total- | Fusiform | L | -40 | -75 | -17 | 3.51 | .496 |

Footnote: +/-: positive/ negative correlations; H = hemisphere; MNI coordinates (x,y,z)

**Supplementary Table 2b: Non-significant (p >.05) whole-brain correlations between gyrification and domains of disgust propensity**

| **Domain** | **Region** | **H** | **X** | **Y** | **Z** | **T** | **p_FWE-corr** |  |
| --- | --- | --- | --- | --- | --- | --- | --- | --- |
| Animal-Reminder+ |  | No suprathreshold clusters | | | | | | |
| Animal-Reminder- | Middle Temporal | L | -62 | -8 | -19 | 3.75 | .169 |  |
|  |  |  |  |  |  |  |  |  |
| Core-Disgust+ |  | No suprathreshold clusters | | | | | | |
| Core-Disgust- | Postcentral | R | 57 | -7 | 40 | 3.68 | .246 |  |
|  |  |  |  |  |  |  |  |  |
| Contamination+ | Calcarine | L | -7 | -90 | 2 | 4.04 | .076 |  |
| Contamination- |  | No suprathreshold clusters | | | | | | |
|  |  |  |  |  |  |  |  |  |
| Total+ |  | No suprathreshold clusters | | | | | | |
| Total- | Cingulum, middle | L | -9 | -36 | 53 | 3.52 | .378 |  |

Footnote: +/-: positive/ negative correlations; H = hemisphere; MNI coordinates (x,y,z)

**Supplementary Table 2c:Non-significant (p >.05) whole-brain correlations between cortical thickness and domains of disgust propensity**

| **Domain** | **Region** | **H** | **X** | **Y** | **Z** | **T** | **p_FWE-corr** |  |
| --- | --- | --- | --- | --- | --- | --- | --- | --- |
| Animal-Reminder+ | Middle Temporal | L | -50 | -9 | -16 | 4.02 | .090 |  |
| Animal-Reminder- |  | No suprathreshold clusters | | | | | | |
|  |  |  |  |  |  |  |  |  |
| Core-Disgust+ | Cingulum, middle | R | 1 | -14 | 28 | 3.25 | .695 |  |
| Core-Disgust- | Precentral | L | -44 | -4 | 47 | 3.60 | .335 |  |
|  |  |  |  |  |  |  |  |  |
| Contamination+ | Cingulum, middle | R | 1 | -23 | 26 | 3.64 | .295 |  |
| Contamination- | Occipital, middle | L | -41 | -77 | 23 | 3.59 | .338 |  |
|  |  |  |  |  |  |  |  |  |
| Total+ | Middle Frontal, orbital | L | -8 | 50 | -6 | 3.26 | .684 |  |
| Total- |  | No suprathreshold clusters | | | | | | |

Footnote: +/-: positive/ negative correlations; H = hemisphere; MNI coordinates (x,y,z)
