# Supplementary material for: The association between local brain structure and disgust propensity
Source: Sci Rep. 2022 Jan 25;12:1327. doi: 10.1038/s41598-022-05407-4 (PMC8789785; doi:10.1038/s41598-022-05407-4)
Supplement: Supplementary file 3 — Supplementary Table 3. [file 41598_2022_5407_MOESM3_ESM.docx]

Table 3: Studies and scanner settings

| **Study** | **Year** | **TR** | **TE** | **FA** | **ST** | **IT** | **Matrix** | **Voxel size** | **Slices** |  |  |
| --- | --- | --- | --- | --- | --- | --- | --- | --- | --- | --- | --- |
| 1 | 2005 | 1900 | 4,15 | 15 | 1 | 1100 | 256*256 | 1 mm x 1 mm x 1 mm | 160 sag. | tfl3d1_ns | Siemens Symphony (1.5 T) |
| 2 | 2006 | 1900 | 4,18 | 15 | 1 | n/a | n/a | 1 mm x 1 mm x 1 mm |  |  |  |
| 3 | 2009 | 1990 | 4,18 | 15 | 1 | 1100 | 256 | 1 mm x 1 mm x 1 mm |  | tfl3d1_ns | Siemens Symphony (1.5 T) |
| 4 | 2015 | 1680 | 1,89 | 8 | 0.88 | 1000 | 256 | 0.88 * 0.88 * 0.88 | 192 | tfl3d1_16ns | Siemens Skyra |
| 5 | 2011 | 1900 | 2,19 | 9 | 1 | 900 | 256 | 1 mm x 1 mm x 1 mm |  | tfl3d1_ns | Siemens Tim Trio (3 T) |
| 6 | 2017 | 1680 | 1,89 | 8 | 0.88 | 1000 | 256 | 0.88 * 0.88 * 0.88 | 192 | tfl3d1_16ns | Siemens Skyra |
| 7 | 2014 | 1560 | 2,07 | 9 | 1 | 900 | 256 | 1 mm x 1 mm x 1 mm |  | tfl3d1_16ns | Siemens Skyra |
| 8 | 2017 | 1680 | 1,89 | 8 | 0.88 | 1000 | 256 | 0.88 * 0.88 * 0.88 | 192 | tfl3d1_16ns | Siemens Skyra |
| 9 | 2014 | 1680 | 1,89 | 8 | 0.88 | 1000 | 256 | 0.88 * 0.88 * 0.88 | 192 | tfl3d1_16ns | Siemens Skyra |
| 10 | 2012 | 1560 | 2,07 | 9 | 1 | 900 | 256 | 1 mm x 1 mm x 1 mm |  | tfl3d1_16ns | Siemens Skyra |
| 11 | 2015 | 1680 | 1,89 | 8 | 0.88 | 1000 | 256 | 0.88 * 0.88 * 0.88 | 192 | tfl3d1_16ns | Siemens Skyra |
| 12 | 2013 | 1300 | 2,69 | 9 | 2 | 900 |  | 0.8*0.8*2 | 104 | n/a | Siemens Tim Trio (3 T) |
